# Guanylate-Binding Protein 1 Promotes Migration and Invasion of Human Periodontal Ligament Stem Cells

**DOI:** 10.1155/2018/6082956

**Published:** 2018-11-28

**Authors:** Shi Bai, Tao Chen, Xia Deng

**Affiliations:** ^1^Stomatological Hospital of Chongqing Medical University, Chongqing 400065, China; ^2^Chongqing Key Laboratory of Oral Diseases and Biomedical Sciences, Chongqing 400044, China; ^3^Chongqing Municipal Key Laboratory of Oral Biomedical Engineering of Higher Education, 401147 Chongqing, China; ^4^Department of Stomatology, Nuclear of Industry 416 Hospital, Chengdu, Sichuan 610051, China

## Abstract

Mesenchymal stem/stromal cells (MSCs) are capable of migrating to sites of injury and inflammation in response to various cytokines to improve tissue repair. Previous studies have shown interferon-gamma (IFN-*γ*) promoted migration of the V54/2 cell line and dental pulp stem cells (DPSCs), but the underlying mechanisms remain largely unknown. In this study, we found IFN-*γ* induced migration and invasion of periodontal ligament stem cells (PDLSCs) in a dose-dependent manner *in vitro*. While knockdown of guanylate-binding protein 1 (GBP1) suppressed IFN-*γ*-induced migration and invasion, ectopic expression of GBP1 potentiated IFN-*γ*-induced migration and invasion of PDLSCs. Furthermore, we demonstrated GBP1 was required for IFN-*γ*-induced processing of matrix metallopeptidase 2 (MMP2) in PDLSCs. Our findings indicate that GBP1 promotes IFN-*γ*-induced migration and invasion of PDLSCs by MMP2, and GBP1 may serve as a new target to facilitate MSC homing and migration.

## 1. Introduction

Mesenchymal stem/stromal cells (MSCs) are a heterogeneous population of cells that are capable of self-renewal and differentiation into specific sublineages, including osteoblasts, adipocytes, and chondrocytes [1, 2]. In addition to their promising potentials in bone and cartilage regeneration, MSCs are currently being investigated in various studies and clinical trials of immunological disorders, such as graft-versus-host disease (GVHD), Crohn's disease, and rheumatoid arthritis [3, 4]. Although administration of exogenous MSCs can be delivered locally and systemically, the migration activity of MSCs to the sites of injury is critical for the therapeutic effects [5, 6]. MSCs are classically isolated from bone marrow, and they can be found in multiple fetal and adult tissues, including umbilical cord blood, fetal lung, and adipose tissue, as well as dental tissues [7, 8]. In dental tissues, MSCs have been found in dental pulp, periodontal ligament, and apical papilla [9–11]. Periodontal ligament stem cells (PDLSCs), a kind of dental stem cells, possess comparable proliferation rate with bone marrow-derived MSCs (BM-MSCs) [10, 12]. In regard to differentiation capacity, PDLSCs can also give rise to osteoblasts, chondrocytes, and adipocytes [10, 12].

Interferon-gamma (IFN-*γ*) plays an important role in innate and adaptive immune responses against infections of various viruses and bacteria [13]. IFN-*γ* also showed critical regulatory effects on multiple biological behaviors of MSC, including proliferation, migration, and osteogenic differentiation. He et al. reported that treatment with low concentration of IFN-*γ* at 0.05 ng/ml can significantly promote the proliferation but inhibit osteogenic differentiation of dental pulp stem cells (DPSCs) *in vitro* [14]. Treatment with IFN-*γ* at 100 IU/ml (approximately 5 ng/ml) inhibited both proliferation and osteogenic differentiation potential of human BM-MSCs [15]. In regard to MSC migration, IFN-*γ* promoted migration of the V54/2 cell line and DPSCs, although the underlying mechanism is still not fully understood [14, 16]. In addition to the continuous exposure to IFN-*γ*, IFN-*γ* was also used for pretreatment of MSCs. Interestingly, IFN-*γ* prestimulation of MSCs increased their migration potential to the inflamed sites and reduced mucosal damage in experimental colitis [17].

Guanylate-binding protein 1 (GBP1) is a type of cytokine-induced guanosine triphosphatase and is an IFN-*γ* response factor [18–20]. GBP1 plays an essential role in mediating the antibacterial and antiviral activities of IFN-*γ* [21, 22]. We have previously shown GBP1 is of highest expression level in all the 7 GBPs in human BM-MSCs, and GBP1 is required for IFN-*γ*-induced processing of *indoleamine 2,3 dioxygenase* (*IDO*), *Interleukin 6* (IL-6), and *IL-8* [23]. Given the critical role of GBP1 in IFN-*γ* signaling, we hypothesized GBP1 may also play an important role in migration of MSCs in response to IFN-*γ* treatment. To address this, we investigated the migration and invasion capacity in GBP1-depleted and GBP1-overexpressed PDLSCs in response to IFN-*γ* treatment, respectively. We further found GBP1 was required for IFN-*γ*-induced upregulation of matrix metalloproteinase 2 (MMP2) expression in PDLSCs.

## 2. Methods and Materials

### 2.1. Subjects and Cell Culture

Disease-free impacted third molars that were indicated for extraction were collected from patients, aged 20–28 years, at Stomatological Hospital of Chongqing Medical University. Written informed consent was obtained from each patient. PDLSCs were isolated as previously described [10]. All procedures were conducted in accordance with the guidelines and regulations approved by Chongqing Medical University. PDLSCs were maintained in DMEM, containing 15% heat-inactivated fetal bovine serum, 100 U/ml of K-Penicillin G and 100 mg/ml of streptomycin sulfate (all from Thermo Fisher Scientific) at 37°C in a humidified atmosphere of 5% CO_2_. PDLSCs within passages 4–10 from up to 4 donors were used in this study. Recombinant human IFN-*γ* (R&D systems) was used to treat PDLSCs as indicated.

### 2.2. siRNA Silencing and Overexpression of GBP1

A pool of 3 target-specific 19–25 nt siRNAs targeting human GBP1 and scrambled siRNAs (siSCR) were purchased from Santa Cruz Biotechnology. PDLSCs were overnight plated, and siRNA transfection was performed using Lipofectamine RNAiMAX reagent (Thermo Fisher Scientific) according to the manufacturer's instructions. For ectopic expression of GBP1, lentiviruses expressing human GBP1 gene were purchased from Fulengen Inc. (Guangzhou, China). PDLSCs were infected in the presence of 8 *μ*g/ml of polybrene (Sigma) for 2 days and then selected with 2 *μ*g/ml puromycin for 3 days. PDLSCs transfected by empty vector were used as control.

### 2.3. Monolayer Cell Wound Healing Assay

The 100% confluent monolayer PDLSCs were scraped with sterile 200 *μ*l pipette tips. The cells were then washed to remove cellular debris and allowed to migrate for 24 hours. Representative images at the initial time point (0 h) and after incubation (24 h) were acquired using an inverted microscope (Olympus) to evaluate the migration distance as previously described [24].

### 2.4. Transwell Assay

1 × 10^5^ PDLSCs in 200 *μ*l of DMEM without FBS were seeded into Matrigel invasion chambers (Corning, NY). Transwell lower chamber is filled with 500 *μ*l of complete growth medium. After 48 hours, the invaded cells were stained with the HEMA-3 kit (Fisher) and counted under optical microscope (Olympus).

### 2.5. RNA Isolation and RT-qPCR

Total RNA was extracted from the cells using the TRIzol reagent according to the manufacturer's instructions (Thermo Fisher Scientific) and treated with RQ1 DNase (Promega). 2 *μ*g aliquots of total RNA was used to generate the first-strand complementary DNA. Quantitative polymerase chain reaction (qPCR) was performed using SYBR Premix Ex Taq kit (TAKARA) using various specific primer sets (Table 1).

### 2.6. Western Blot

Protein was isolated using RIPA buffer (Santa Cruz Biotechnology) containing 1% protease inhibitor cocktail (Roche) according to the manufacturer's instructions. 25–40 *μ*g aliquots of the lysates were separated on a 10% sodium dodecyl sulfate-polyacrylamide gel. The resolved proteins were transferred onto nitrocellulose membrane (Bio-Rad) and incubated with antibodies overnight at 4°C. Protein bands were detected using an enhanced chemiluminescence Western blotting detection kit (Thermo Fisher Scientific). Antibodies used in this study includes rabbit polyclonal anti-human GBP1 antibody (Invitrogen) and rabbit polyclonal anti-human MMP2 antibody (Cell Signaling Technology).

### 2.7. Statistical Analysis

Student's *t*-test were used for single comparisons. One-way analysis of variance (ANOVA) and two-way ANOVA with post-hoc Tukey's tests were performed for multiple comparisons using GraphPad Prism 6 software. Data was expressed as mean ± SD from at least three independent experiments. *p* < 0.05 was considered as significant. ^∗^ *p* < 0.05; ^∗∗^ *p* < 0.01; ^∗∗∗^ *p* < 0.001.

## 3. Results

### 3.1. IFN-*γ* Treatment Promoted Migration and Invasion and Expression of GBP1 in a Dose-Dependent Manner in PDLSCs

**Figure 1:**
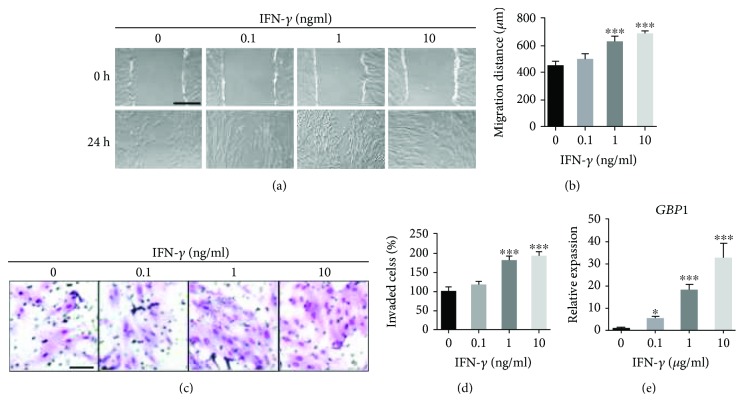
IFN-*γ* promoted migration and invasion and expression of GBP1 in PDLSCs. Results of wound healing assay revealed an increase in migration distance of PDLSCs in response to IFN-*γ* treatment at 1 and 10 ng/ml (a and b). Bar indicates 500 *μ*m in (a). Results of transwell assay showed an increase in invade cell number of PDLSCs in response to IFN-*γ* treatment at 1 and 10 ng/ml (c and d). Bar indicates 100 *μ*m in (c). GBP1 expression was upregulated in IFN-*γ*-treated PDLSCs as determined by RT-qPCR (e). ^∗^ *p* < 0.05; ^∗∗∗^ *p* < 0.001.

To investigate the effect of IFN-*γ* on migration of PDLSCSs, monolayer cell wound healing assay was performed. As shown in Figures 1(a) and 1(b), the migration distance of PDLSCs was significantly increased in response to IFN-*γ* treatment at 1 and 10 ng/ml but not at 0.1 ng/ml. Consistently, the invasion capacity of PDLSCs was also enhanced by IFN-*γ* treatment at 1 and 10 ng/ml but not at 0.1 ng/ml (Figures 1(c) and 1(d)). We further confirmed that the expression level of GBP1, a well-established IFN-*γ* response gene, was induced by IFN-*γ* treatment in a dose-dependent manner in PDLSCs (Figure 1(e)). As 10 ng/ml was the optimal concentration for IFN-*γ*-induced migration and invasion and expression of *GBP1* in PDLSCs, this concentration was selected for the rest of the study.

### 3.2. siRNA-Mediated Depletion of GBP1 Suppressed IFN-*γ*-Induced Migration and Invasion of PDLSCs

**Figure 2:**
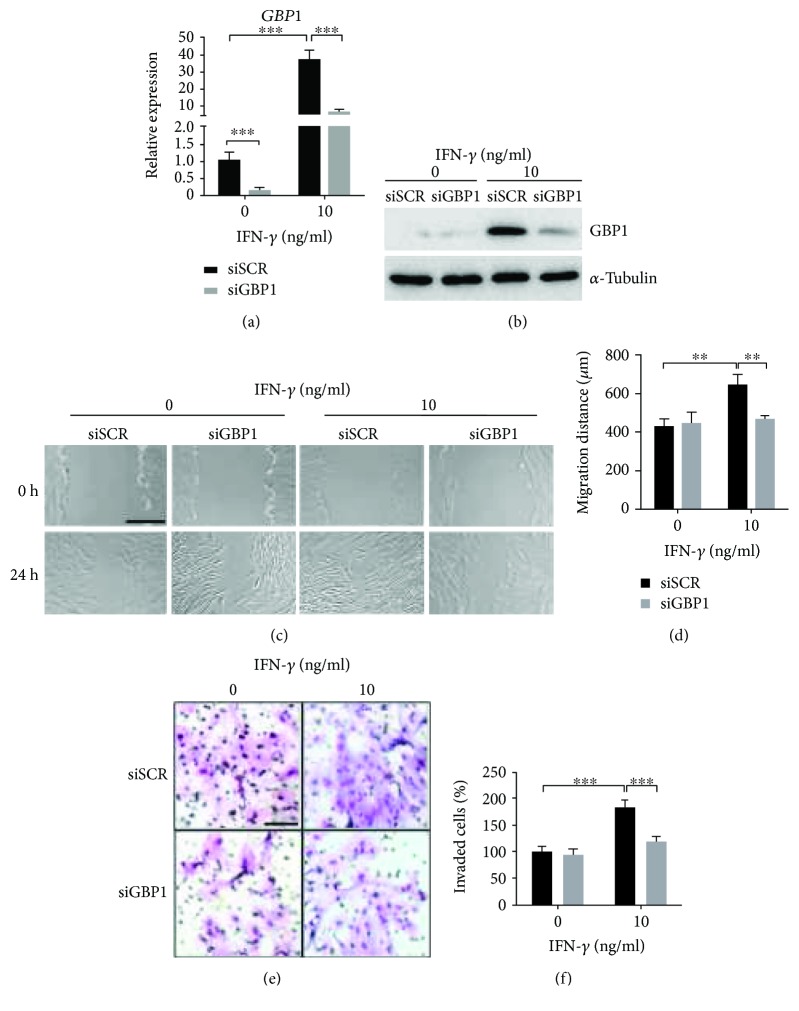
Depletion of GBP1 suppressed IFN-*γ*-induced migration and invasion of PDLSCs. The knockdown efficiency was confirmed by RT-qPCR and Western blot (a and b). To examine the effect of GBP1 knockdown on migration of PDLSCs, wound healing assay was performed using GBP1-depleted PDLSCs (siGBP1) and scrambled siRNA-transfected cells (siSCR). Representative images were acquired at the initial time point (0 h) and after 24-hour migration (24 h), respectively. The upregulation of migration induced by IFN-*γ* treatment was inhibited by GBP1 knockdown (c and d). Bar indicates 500 *μ*m in (c). The upregulation of invasion induced by IFN-*γ* treatment was also inhibited by GBP1 knockdown as revealed by transwell invasion assay (e and f). Bar indicates 100 *μ*m in (e). ^∗∗^ *p* < 0.01; ^∗∗∗^ *p* < 0.001.

Next, we sought to investigate whether GBP1 is required for IFN-*γ*-induced migration and invasion. A pool of siRNAs targeting GBP1 was used to knockdown GBP1 expression, and the knockdown efficiency was confirmed by RT-qPCR analysis and Western blot (Figures 2(a) and 2(b)). The transfected cells were then seeded into six-well plates and allowed to grow confluent. Interestingly, depletion of GBP1 significantly inhibited the IFN-*γ*-induced upregulation of migration activity, while it did not affect the migration of PDLSCs in absence of IFN-*γ* (Figures 2(c) and 2(d)). Depletion of GBP1 also suppressed the increase in invaded cell number induced by IFN-*γ* treatment at a concentration of 10 ng/ml (Figures 2(e) and 2(f)).

### 3.3. Overexpression of GBP1 Potentiated IFN-*γ*-Induced Migration and Invasion of PDLSCs

**Figure 3:**
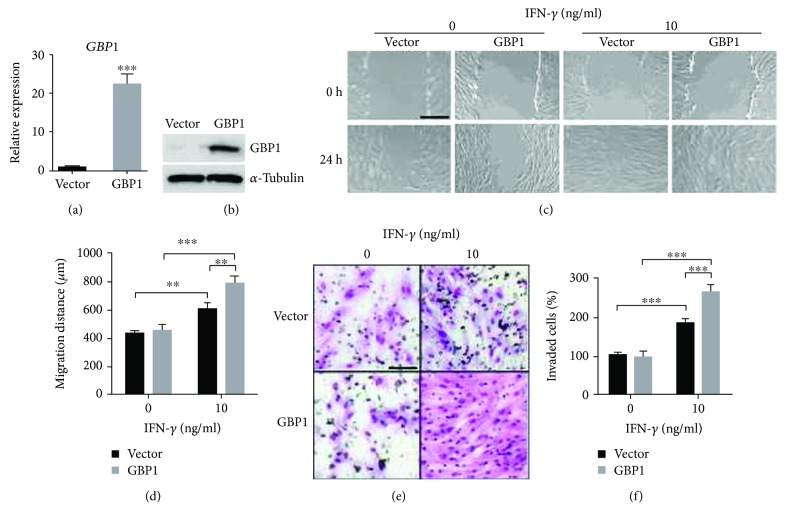
Overexpression of GBP1 potentiated IFN-*γ*-induced migration and invasion of PDLSCs. The overexpression of GBP1 in PDLSCs was confirmed by RT-qPCR and Western blot (a and b). Wound healing assay was performed using GBP1-overexpressed PDLSCs and control cells (vector). Representative images were acquired at the initial time point (0 h) and after 24-hour migration (24 h), respectively. Overexpression of GBP1 further enhanced the migration as well as invasion of PDLSCs induced by IFN-*γ* treatment (c–f). Bars indicate 500 *μ*m in (c) and 100 *μ*m in (e), respectively. ^∗∗∗^ *p* < 0.001.

Next, PDLSCs were infected by lentiviruses expressing GBP1, and the overexpression of GBP1 was confirmed by RT-qPCR analysis and Western blot (Figures 3(a) and 3(b)). As shown in Figures 3(c) and 3(d), ectopic expression of GBP1 did not promote the migration of PDLSCs, but it further enhanced the migration activity of PDLSCs in presence of IFN-*γ* at a concentration of 10 ng/ml. The invasion capacity of IFN-*γ*-treated PDLSCs was also promoted by ectopic expression of GBP1 (Figures 3(e) and 3(f)). Above all, these findings indicated GBP1 is essential for the upregulation of migration and invasion of PDLSCs induced by IFN-*γ* treatment.

### 3.4. GBP1 Was Required for IFN-*γ*-Induced Processing of MMP2 in PDLSCs

**Figure 4:**
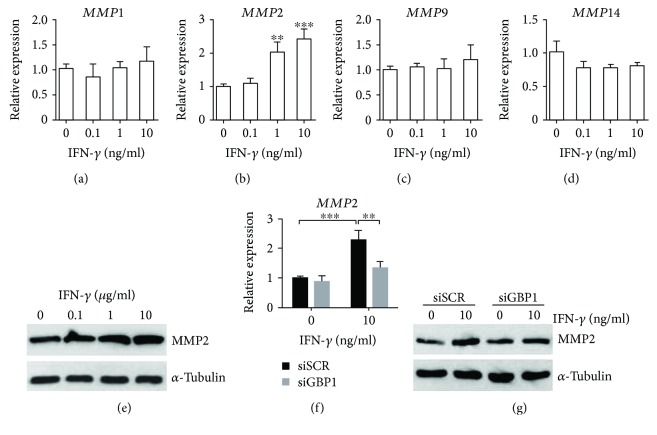
GBP1 is required for upregulation of MMP2 by IFN-*γ* treatment in PDLSCs. RT-qPCR analysis of matrix metalloproteinase gene expression after 48 hours of IFN-*γ* treatment in PDLSCs and control cells (a–d). MMP2 expression was upregulated by IFN-*γ* treatment in a dose-dependent manner (b and e). The IFN-*γ*-induced upregulation of MMP2 was inhibited by depletion of GBP1 as determined by RT-qPCR analysis and Western blot (f and g). ^∗∗^ *p* < 0.01; ^∗∗∗^ *p* < 0.001.

Previous studies have reported that MMPs play an important role in regulating the migratory activity of MSCs [25, 26]. We screened the expression levels of several MMPs by RT-qPCR in PDLSCs in response to IFN-*γ* treatment for 48 hours. Interestingly, IFN-*γ* treatment specifically induced *MMP2* expression in PDLSCs, instead of *MMP1*, *MMP9*, and *MMP14* (Figures 4(a)–4(d)). We also investigated the effect of IFN-*γ* treatment on expression of carcinoembryonic antigen-related cell adhesion molecule 1 (CEACAM1) and intercellular adhesion molecule 1 (ICAM1), and we found IFN-*γ* treatment did not affect the mRNA expression of *CEACAM1* or *ICAM1* in PDLSCs (Supplementary Figure 1 (a and b)). We then confirmed the upregulation of MMP2 by IFN-*γ* treatment in PDLSCs by Western blot (Figure 4(e)). Furthermore, we found depletion of GBP1 significantly suppressed IFN-*γ*-induced processing of MMP2 (Figures 4(f) and 4(g)). Taken together, our findings suggested GBP1 is required for IFN-*γ*-induced processing of MMP2 thereby promoting migration and invasion of PDLSCs induced by IFN-*γ* treatment.

## 4. Discussion

In this study, we found treatment with IFN-*γ* promoted migration and invasion of PDLSCs, and enhanced expression levels of MMP2. Although depletion of GBP1 did not affect the migration and invasion capacity of PDLSCs, it significantly suppressed the upregulation of migration and invasion induced by IFN-*γ* treatment. In addition, ectopic expression of GBP1 in PDLSCs further enhanced the IFN-*γ*-induced migration and invasion of PDLSCs. Finally, we showed GBP1 was required for the processing of MMP2 induced by IFN-*γ*, by which it may promote the IFN-*γ*-induced migration and invasion of PDLSCs.

The migration activity of MSCs to the sites of injury is critical for their function to facilitate tissue repair. Previous studies have shown IFN-*γ* may promote migration of MSCs, depending on the treatment concentration and the source of MSCs. Treatments with IFN-*γ* at 0.05, 0.5, and 5 ng/ml enhanced migration activity of DPSCs [14], and treatments with IFN-*γ* at 1000 U/mL (approximately 50 ng/ml) promoted migration activity in V54/2 cell line. However, treatment with IFN-*γ* at 1000 IU/ml (approximately 50 ng/ml) only slightly increased the migration of BM-MSCs, but only one concentration level of IFN-*γ* was tested in that study [16]. In this study, we found the migration and invasion of PDLSCs was significantly increased in response to IFN-*γ* treatment at 1 and 10 ng/ml. More importantly, our findings indicate GBP1 is a pivotal mediator in IFN-*γ*-induced migration of PDLSCs. Since the optimal concentration of IFN-*γ* might vary across individuals, it is possible that targeting GBP1 is more practicable for clinical applications. Furthermore, the immune properties of MSCs after homing to the injured sites are also important for tissue repair. Although recent studies suggest resting MSCs may not have significant immunomodulatory activity, but treatment with IFN-*γ* are capable of enhancing the immunosuppressive properties of MSCs. For instance, MSCs primed with IFN-*γ* was found to prevent the development of GVHD more efficiently, compared to unstimulated MSCs [27, 28]. Since GBP1 is essential for IFN-*γ* signaling, GBP1 may also play a role in IFN-*γ*-induced cytokine production and immunomodulatory activity of MSCs [16, 23].

MMPs are a family of zinc-dependent proteolytic enzymes that are involved in the degradation of extracellular matrix (ECM). Previous studies have highlighted the role of MMP1, MMP2, MMP9, and MMP14 in regulation of MSC migration [25, 26, 29]. Expression of MMP2 and MMP9 was elevated by IFN-*γ* treatment in a human salivary gland cell line (HSG), but IFN-*γ* inhibits constitutive MMP-2 expression in human astroglioma cells [30, 31]. In this study, we found IFN-*γ* treatment specifically induced expression of *MMP2*, instead of *MMP1*, *MMP9*, and *MMP14* in PDLSCs, and GBP1 was required for IFN-*γ*-induced processing of MMP2 as evidenced by RT-qPCR and Western blot. In addition, He et al. reported IFN-*γ* regulates cell behavior of DPSCs via NF-*κ*B and MAPK signaling. More efforts are still needed to investigate how GBP1 is incorporated into the downstream signaling in IFN-*γ*-treated MSCs. Duijvestein *et al.* have showed *CEACAM1* expression was upregulated after 6 days of treatment with IFN-*γ* in human BM-MSCs [17]. However, in this study, we found 2 days of IFN-*γ* treatment did not affect the mRNA expression of *CEACAM1* or *ICAM1* in PDLSCs. It is possible that *CEACAM1* is not direct target of IFN-*γ* signaling, but it can be induced by activation of NF-κB and/or MAPK signaling.

In conclusion, our findings indicate GBP1 mediates IFN-*γ*-induced migration of PDLSCs via MMP2. And GBP1 may be a new therapeutic target to promote MSC migration that can facilitate treatment with IFN-*γ*-primed MSCs.

## Figures and Tables

**Table 1:** Primer sequence.

Gene	Forward primer (5′-3′)	Reverse primer (5′-3′)
*GAPDH*	GGAGCGAGATCCCTCCAAAAT	GGCTGTTGTCATACTTCTCATG
*GBP1*	GAAGTGCTAGAAGCCAGTGC	CCACCACCATAGGCTGTGTA
*MMP1*	ATGAAGCAGCCCAGATGTGGAG	TGGTCCACATCTGCTCTTGGCA
*MMP2*	AGCGAGTGGATGCCGCCTTTAA	CATTCCAGGCATCTGCGATGAG
*MMP9*	TGTACCGCTATGGTTACACTCG	GGCAGGGACAGTTGCTTCT
*MMP14*	CCTTGGACTGTCAGGAATGAGG	TTCTCCGTGTCCATCCACTGGT
*CEACAM1*	AGCTCATGGACCTTTCCAGAC	GTTTCCTGCGCTGTCGTTTG
*ICAM1*	ATGCCCAGACATCTGTGTCC	GGGGTCTCTATGCCCAACAA

## Data Availability

The data used to support the findings of this study are included within the article.

## Supplementary Materials

Supplementary Materials The effect of IFN-*γ* treatment on expression of *CEACAM1* or *ICAM1* in PDLSCs. Supplementary Figure 1: IFN-*γ* treatment did not affect the expression of CEACAM1 or ICAM1 in PDLSCs. RT-qPCR analysis of *CEACAM1* expression after 48 hours of IFN-*γ* treatment in PDLSCs and control cells (a). RT-qPCR analysis of *ICAM1* expression after 48 hours of IFN-*γ* treatment in PDLSCs and control cells (b).
