# Supplementary figures and images for: Guanylate-Binding Protein 1 Promotes Migration and Invasion of Human Periodontal Ligament Stem Cells
Source: Stem Cells Int. 2018 Nov 28;2018:6082956. doi: 10.1155/2018/6082956 (PMC6304207; doi:10.1155/2018/6082956)

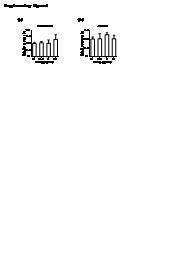

Supplement: Supplementary Materials — The effect of IFN-γ treatment on expression of CEACAM1 or ICAM1 in PDLSCs. Supplementary Figure 1: IFN-γ treatment did not affect the expression of CEACAM1 or ICAM1 in PDLSCs. RT-qPCR analysis of CEACAM1 expression after 48 hours of IFN-γ treatment in PDLSCs and control cells (a). RT-qPCR analysis of ICAM1 expression after 48 hours of IFN-γ treatment in PDLSCs and control cells (b). [file 6082956.f1.xps › docProps/thumbnail.jpeg]
